# Factors Related to Attempted Suicide in Davanagere

**DOI:** 10.4103/0970-0218.39237

**Published:** 2008-01

**Authors:** MR Nagendra Gouda, Sambaji M Rao

**Affiliations:** Basaveshwara Medical College and Hospital, Chitradurga, India; 1 J.J.M. Medical College, Davanagere, Karnataka, India

**Keywords:** Suicide in India, attempted suicide, factors

## Abstract

**Research Question:**

What are the factors responsible for suicidal attempts?

**Objectives:**

To study the socio-demographic factors, methods and reasons for suicidal attempts.

**Type of Study:**

Cross-sectional study.

**Setting:**

Bapuji and C.G. Hospitals attached to J.J.M. Medical College, Davanagere.

**Participants:**

A total of 540 suicidal attempters admitted to emergency wards.

**Methodology:**

A pretested proforma was administered to the subjects relating the factors responsible for the attempt. The data thus obtained was compiled and analyzed.

**Statistical Analysis:**

Proportions, *Z*-test and Chi-square test.

**Results:**

In this study, 61.3% were males and 38.7% were females. Peak occurrence of suicidal attempts was found in the second and third decades (15-29 years). Hindus constituted about 94.6% of the total suicidal attempters. Almost half (52.2%) of the subjects had education below or up to matriculation and 83% of them were from the lower (classes IV and V) socio-economic groups. Agriculturists, housewives and unskilled workers represented 75% of the total subjects. Fifty-five percent of the subjects were from nuclear families and most (62.4%) of them were married; frequent mode of attempting suicides was by organo-phosphorus compounds (66.3%) followed by overdosage of tablets (17.8%). Common cause was family problem (27.2%) followed by illness (27%).

A high suicide rate in any society is an index of social disorganization. World Health Report 2001 estimates that 10-20 million people make an attempt to commit suicide and 1 million people become successful in it. The comparable period prevalence rate for suicide throughout the world ranges from 5 to 30/lakh populations per year.(1) One million people committed suicide in 2000 worldwide. Hence, like other non-communicable diseases, suicide is increasing in trend throughout the world and is a major public health problem.(2) Suicide is one of the 10 major causes of deaths in India. India ranks second in number of suicidal deaths.(3) According to National Crime Records Bureau (2001), there were 108,506 deaths due to suicide, with an incidence rate of 11 per one lakh population during 2000. In 1974, the number of suicide cases was 46,008, which have now tripled in the past three decades,(4) reflects the seriousness of the problem.

Suicide is a result of complex interaction between biological, psychological, social and situational factors. Similarly, attempted suicide is stated to be associated with several psychosocial and medical conditions, viz, young age of 15-24 years, female sex, poor education, unemployment, living alone and history of socio-economic deprivation are stated to be potential risk factors.(5) However, studies have failed to reveal consistent findings with regard to the prevalence and potency of these risk factors in different regions. At times, suicide appears to be a very private affair with problems or burdens not shared even with the closest people of the attempter. Therefore, there is a need to identify such factors associated with the suicidal attempt.

A number of studies have been done all over the world and in India in order to find out the reasons that make life miserable or worthless. It is not known whether the risk factors reported in studies from the Western countries can be applied to our country. Therefore, there is a need to identify the risk factors associated with the suicidal attempt pertaining to our cultural norms.

## Materials and Methods

This cross-sectional study was conducted at Bapuji and C.G. Hospitals, two teaching hospitals, attached to J.J.M. Medical College, Davanagere. Davanagere is located in central part of Karnataka. Apart from the same district, these hospitals also cater patients from the neighbouring districts. This study was conducted for a period of 1 year starting from 1 April 2004 to 31 March 2005. For the purpose of the study, a case of suicidal attempt was defined as: “A person who had made deliberate act of self-harm consciously aimed at self-destruction, irrespective of his/her intention to die”.

A total of 756 patients admitted to the emergency wards of these hospitals were contacted about the participation in the study. A total number of 216 attempters were excluded from the study either due to refuse to take part in the study or discharged before the interrogation. Remaining 540 attempters gave their consent and constituted the study group. They were interviewed by using a pretested proforma. The data thus obtained was compiled and analyzed by using SPSS 13 (Statistical Package for Social Sciences, Version 13).

## Results

Of the 540 suicidal attempters studied, 61.3% were males and 38.7% were females, which gave a male-to-female ratio of 1.6:1. The mean age (±SD) of male attempters was (30.41 ± 10.18 years) significantly higher than that of females (27.26 ± 11.44 years; *Z* = 3.3, *P* < 0.001). The youngest attempters were of 15 years and oldest were of 70 years. Peak occurrence of suicidal attempts was found in the second and third decades (15-29 years) and it was least in the sixth decade, especially in females. More number of males attempted suicide in 20- to 39-year age group. The age-specific attempted suicidal rates decreased with increase in both the sexes [Table 1].

**Table 1:** Age and sex distribution of the study group

Age group (year)	Sex *N* (%)		Total *N* (%)
		
	Male	Female	
15-19	31 (9.4)	51 (24.4)	82 (15.2)
20-24	84 (25.4)	60 (28.7)	144 (26.7)
25-29	63 (19.0)	32 (15.3)	95 (17.6)
30-34	38 (11.5)	15 (7.2)	53 (9.8)
35-39	55 (16.6)	20 (9.6)	75 (13.9)
40-44	20 (6.0)	8 (3.8)	28 (5.2)
45-49	22 (6.6)	8 (3.8)	30 (5.6)
50-59	11 (3.3)	9 (4.3)	20 (3.7)
60 and above	7 (2.1)	6 (2.9)	13 (2.4)
Total	331 (100.0)	209 (100.0)	540 (100.0)

Hindus constituted about 94.6% of the total suicidal attempters and only few (5.4%) were Muslims. This may be due to the large Hindu population residing in this area. Approximately 27.4% of the subjects had not received any education. Almost half (52.2%) of suicidal attempters had education below or up to matriculation. The proportion of females with education up to college and above was more (23.4%) compared to males (18.4%) [Table 2].

**Table 2:** Socio-demographic factors of suicidal attempters

	Sex	Male *N* (%)	Female *N* (%)	Total *N* (%)
Religion	Hindu	310 (93.7)	201 (96.2)	511 (94.6)
	Muslim	21 (6.3)	8 (3.8)	29 (5.4)
Education	Uneducated	94 (28.4)	54 (25.8)	148 (27.4)
	School	176 (53.2)	106 (50.7)	282 (52.2)
	College and above	61 (18.4)	49 (23.4)	110 (20.4)
Socio-economic status	Class I	6 (1.8)	12 (5.7)	18 (3.3)
	Class II	21 (6.3)	10 (4.8)	31 (5.7)
	Class III	22 (6.6)	22 (10.5)	44 (8.1)
	Class IV	92 (27.8)	44 (21.1)	136 (25.2)
	Class V	190 (57.4)	121 (57.9)	311 (57.6)
Occupation	Agriculture	145 (43.8)	-	145 (26.9)
	Housewives	-	113 (54.1)	113 (20.9)
	Unskilled	103 (31.1)	40 (19.1)	143 (26.5)
	Skilled	51 (15.4)	3 (1.4)	54 (10.0)
	Students	6 (1.8)	35 (16.7)	41 (7.6)
	Professional	9 (2.7)	13 (6.2)	22 (4.1)
	Semiskilled worker	8 (2.4)	3 (1.4)	11 (2.0)
	Unemployed	9 (2.7)	2 (1.0)	11 (2.0)
Type of family	Nuclear	182 (55.0)	116 (55.5)	298 (55.2)
	Joint	114 (34.4)	65 (31.1)	179 (33.1)
	Three-generation	35 (10.6)	28 (13.4)	63 (11.7)
	Total	331 (100)	209 (100)	540 (100)
Domicile	Rural	230 (60.4)	151 (39.6)	381 (100)
	Urban	101 (63.5)	58 (36.5)	159 (100)
	Total	331 (61.3)	209 (38.7)	540 (100)

Most (83%) of the suicidal attempters were from the lower (classes IV and V) socio-economic groups. The number of suicidal attempters increased gradually with decrease in socio-economic status in both the sexes. This difference was statistically significant (χ^2^ = 11.16, df = 4, *P* < 0.05). The total sample comprised about 75% of agriculturists, housewives and unskilled workers; 54.1% of the total females were housewives. More number of female students and employed females attempted suicide than males. Majority of the suicidal attempters (55%) were from nuclear families, about 33.1% were from joint families and 11.7% of them were from three-generation families. About 70.6% of the suicidal attempters represented rural areas compared to urban population (29.4%).

Majorities (62.4%) of the attempters were married, 33.9% were unmarried and 2.4% of them were widows or widowers. The proportion of married and unmarried male attempters was 60.4% and 38.1%, respectively and in females it was 65.6% and 27.3%, respectively. The difference was statistically significant (χ^2^ = 16.1, df = 3, *P* < 0.001).

Most frequent mode of attempting suicides was by organo-phosphorus compounds (66.3%) followed by overdosage of tablets (17.8%). Other modes of attempting suicide [Table 3] were self-poisoning by other substances, hanging, burns, drowning and fall from height. A difference was noticed in the mode of the attempts: with females preferring overdosage of medications (28.7%) compared to males (10.9%).

**Table 3:** Precipitating factors for the attempt

Precipitating factor for the attempt	Sex			Male vs female *P*-value
		
	Male *N* (%)	Female *N* (%)	Total *N* (%)	
Family problem	91 (27.5)	56 (26.8)	147 (27.2)	NS
Illness	77 (23.3)	69 (33.0)	146 (27.0)	0.013
Financial problem	70 (21.1)	7 (3.3)	77 (14.3)	0.001
School-related problems	9 (2.7)	28 (13.4)	37 (6.9)	0.0001
Love failure	17 (5.1)	4 (1.9)	21 (3.9)	NS
Unemployment	18 (5.4)	-	18 (3.3)	0.001
Crops damage	16 (4.8)	-	16 (3.0)	0.001
Marital disharmony	3 (0.9)	7 (3.3)	10 (1.9)	0.04
Dowry problem	1 (0.3)	7 (3.3)	8 (1.5)	0.004
Fall in social reputation	3 (0.9)	5 (2.4)	8 (1.5)	NS
Alcohol-related problems	3 (0.9)	3 (1.4)	6 (1.1)	NS
Pregnancy-related problems	-	6 (2.9)	6 (1.1)	-
Others	23 (6.9)	17 (8.13)	33 (6.1)	NS
Total	331 (100)	209 (100)	540 (100)	

On analyzing the causes of the suicidal attempts, i.e. the immediate and most significant factor held responsible for the suicidal act, most common cause was family problem (27.2%) followed by illness (27%). Family problem (27.5%) topped the list in males and illness (33%) in females.

## Discussion

In this study, more number of males attempted suicides than females. This is supported by many Indian studies.(6–8) Studies done with respect to the peak occurrence of suicidal attempts had shown increased incidence in the second and third decades of life. These findings confirm that attempted suicides are rising rapidly among the youths. Contrary to these results, a study in China(9) has reported the peak occurrence of suicides in third and fourth decades.

Religion has long been regarded as an important factor in suicide and attempted suicide. Research has shown that suicide rates are more in countries where religious practices are prohibited or strongly discouraged and where Buddhism, Hinduism or the Asian religions predominate.(7,10) Since studies in India were conducted in Hindu-dominated areas, it is difficult to interpret the religious aspect of the suicidal attempt. Muslim nations report lower rates throughout the world, including the Muslim majority in Kashmir.(11)

Low education is an important risk factor for suicide. The individuals with higher educational levels and employed as professionals and semi-professionals constitute less number of suicides and attempted suicides. The findings of the present study are in agreement with the findings,(6,8,10,12) which conclude that low level of education is an important risk factor for suicidal attempt. The number of suicidal attempters increased gradually with decrease in socio-economic status in both the sexes. Most of the studies in different countries have reported that lower social class is an important risk factor for suicide and attempted suicide. Many studies(7,8,10) also observed the same in corroboration with this study results. Low education, less income levels, belonging to unorganized labour sector in the midst of a burgeoning private industrial growth and globalization place a large number of individuals at a high risk of economic insecurity and suicidal behaviour.(7) The more demanding nature of nuclear families, coupled with stress and strains, adds fuel to the fire. There is no one to shoulder their agony. This may drive people to attempt suicide.(10) Reasons for the higher rates of suicidal attempts in many rural areas may include social isolation, easy availability of pesticides, greater difficulty in identifying warning signs, limited access to health facility and doctors, and lower levels of education. Methods of suicide in rural areas are also often different from those used in urban areas.(2) In contrary to these findings, two studies(8,10) noted most of the suicidal attempters were urban dwellers.

WHO reports divorced, widowed and single people are at a higher risk of suicide than married people. The quoted reason was that marriage in India is a social obligation, which is performed by the elders, irrespective of the individual's preparedness for it. Marital partners in India are virtually strangers to each other (due to arranged marriages) and so are the families. Hence, several adjustment issues could arise among the married persons. The findings of this study were also observed in a similar study conducted in Bangalore,(7) whereas other studies(6,8,13–15) observed more number of attempts in unmarried, divorced and widowed persons.

The main mode of attempting suicide was organo-phosphorous compounds in this study, followed by self-poisoning with overdosage of drugs and other common household substances. Similar observations were reported by many studies.(6,8,13–15)

Family conflicts were the major causes for attempting suicides. Subjects with physical and mental illness, financial problems, unemployment, failure in examination were other causes. Similar results were observed in two studies,(6,13) whereas other studies(12,15) noted unemployment as the major cause for attempting suicide.

## Conclusions

Suicides and attempted suicides are slowly but steadily assuming the levels of a public health problem caused by multiple factors. The factors responsible for attempted suicides were in the age group of 15-44 years, male sex, low education level, low socio-economic status, illness and family problems. Among these factors, most of them are preventable and controllable.

The main limitation of this study is that it is a hospital-based cross-sectional study. Community-based longitudinal studies can reveal some more factors and avoid the selection bias. Some cases have been excluded from the study, which could not be accounted. Other risk factors influencing suicidal attempt have not been taken into account.

However, the suicidal attempters represent the “tip of iceberg”. Effective suicide preventive and control measures need to be taken in the form of early identification of suicide-prone individuals. There is an urgent need to institute a national suicide surveillance policy. Micro-level analysis of suicides and suicidal attempts are required to identify high risk population. Apart from strengthening poverty alleviation programmes, inputs from Department of Community Medicine in medical colleges are required in sociology, mental health and community health development. Local NGOs can make a significant contribution in this direction.
